# Integration of the Butina algorithm and ensemble learning strategies for the advancement of a pharmacophore ligand-based model: an *in silico* investigation of apelin agonists

**DOI:** 10.3389/fchem.2024.1382319

**Published:** 2024-04-16

**Authors:** Xuan-Truc Dinh Tran, Tieu-Long Phan, Van-Thinh To, Ngoc-Vi Nguyen Tran, Nhu-Ngoc Song Nguyen, Dong-Nghi Hoang Nguyen, Ngoc-Tam Nguyen Tran, Tuyen Ngoc Truong

**Affiliations:** ^1^ Faculty of Pharmacy, University of Medicine and Pharmacy at Ho Chi Minh City, Ho Chi Minh City, Vietnam; ^2^ Bioinformatics Group, Department of Computer Science, and Interdisciplinary Center for Bioinformatics, Universität Leipzig, Leipzig, Germany; ^3^ Department of Mathematics and Computer Science, University of Southern Denmark, Odense, Denmark; ^4^ Faculty of Pharmacy, Uppsala University, Uppsala, Sweden

**Keywords:** 3D pharmacophore model, APJ receptor agonist, butina clustering algorithm, ensemble learning method, drug discovery

## Abstract

**Introduction:** 3D pharmacophore models describe the ligand’s chemical interactions in their bioactive conformation. They offer a simple but sophisticated approach to decipher the chemically encoded ligand information, making them a valuable tool in drug design.

**Methods:** Our research summarized the key studies for applying 3D pharmacophore models in virtual screening for 6,944 compounds of APJ receptor agonists. Recent advances in clustering algorithms and ensemble methods have enabled classical pharmacophore modeling to evolve into more flexible and knowledge-driven techniques. Butina clustering categorizes molecules based on their structural similarity (indicated by the Tanimoto coefficient) to create a structurally diverse training dataset. The learning method combines various individual pharmacophore models into a set of pharmacophore models for pharmacophore space optimization in virtual screening.

**Results:** This approach was evaluated on Apelin datasets and afforded good screening performance, as proven by Receiver Operating Characteristic (AUC score of 0.994 ± 0.007), enrichment factor of (EF1% of 50.07 ± 0.211), Güner-Henry score of 0.956 ± 0.015, and F-measure of 0.911 ± 0.031.

**Discussion:** Although one of the high-scoring models achieved statistically superior results in each dataset (AUC of 0.82; an EF1% of 19.466; GH of 0.131 and F1-score of 0.071), the ensemble learning method including voting and stacking method balanced the shortcomings of each model and passed with close performance measures.

## 1 Introduction

Apelin (APJ) is a bioactive peptide initially discovered in bovine stomach extracts by Tatemoto et al., in 1998 (Tatemoto et al., 1998). It is acknowledged as the inherent ligand of the human G protein-coupled receptor APJ (APLNR) and a seven-transmembrane receptor akin to the angiotensin-type 1 receptor (Tatemoto et al., 1998). Apelin is a precursor protein composed of 77 amino acids that undergo hydrolysis to yield active peptides of varying lengths, including Apelin-36, Apelin-31, Apelin-17, and Apelin-13. The APJ system is instrumental in the physiological and pathological functioning of numerous organs, influencing fluid homeostasis, blood pressure, cardiac contractility, angiogenesis, metabolic equilibrium, cell proliferation, apoptosis, and inflammation (Hanna Antushevich, 2018). Furthermore, the APJ system exhibits extensive expression in the central nervous system, predominantly in neurons and oligodendrocytes (Wan et al., 2022).

In accordance with the International Union of Pure and Applied Chemistry (IUPAC), a pharmacophore is characterized as the aggregate of steric and electronic attributes that are requisite to guarantee the optimal supra-molecular interactions with a specific biological target structure, thereby inducing (or inhibiting) its biological response (Seidel et al., 2019). The underlying premise of pharmacophoric modeling is that the presence of common chemical functionalities, coupled with a similar spatial arrangement, would culminate in biological activity on the same target. The most critical pharmacophoric feature types encompass hydrogen bond acceptors, hydrogen bond donors, hydrophobic regions, positively and negatively ionizable groups, aromatic groups, and metal coordinating areas. Pharmacophore models can be classified into two primary categories: ligand-based models and structure-based models.

The ligand-based 3D-pharmacophore approach, extensively utilized in virtual drug screening, constructs predictive models from active datasets, drawing upon the study of 3D configurations and interactions among functional molecules (Giordano et al., 2022). Nevertheless, the efficacy of pharmacophore models is intrinsically tied to the diversity and precision of the input training dataset. Consequently, data clustering is implemented via the Butina algorithm to amass the centroids prior to the construction of pharmacophore models in pharmacophore elucidator - MOE 2015.10 (Vilar et al., 2008). The centroids derived from each active cluster are collated to formulate a structural training dataset, while the remaining active compounds are utilized in decoy generation by the DeepCoy model (Imrie et al., 2021). DeepCoy fabricates high-caliber decoys that pose a challenge to differentiate from active substances, thereby mitigating the risk of artificial enrichment, analog bias, and false negative bias. Artificial enrichment pertains to the performance influenced by the disparities in chemical space between the active and decoy molecules. Analog bias emerges from the restricted diversity of the active molecules, while false negative bias refers to the potential presence of active compounds within the decoy set, which could result in an underestimation of the screening performance.

Ensemble learning (Rokach, 2010) serves as an exhaustive meta-approach in machine learning, striving to augment predictive performance via the integration of predictions derived from an array of models. Despite the seemingly boundless ensemble configurations that can be tailored for a predictive modeling problem, there are three methods that primarily preside over the domain of ensemble learning. Each of these methods extends beyond being a mere algorithm, evolving into a distinct field of study that has engendered a multitude of specialized methods. The four cardinal categories of ensemble learning methods comprise voting, bagging, stacking, and boosting. It is imperative to not only acquire a profound comprehension of each method but also to contemplate their incorporation in predictive modeling project.

Drawing upon the research conducted by Wieder et al. (Wieder et al., 2017), which led to the development of the common hits approach method, a technique akin to the voting method, and the work of Kumar et al. (Kumar, 2018), who created the REPHARMBLE tools that employ Poisson statistics and entropy calculations based on information theory, we have devised ensemble methods (voting and stacking) for the development of ligand-based pharmacophore models. These models diverge from the structure-based research of these studies and incorporate the use of machine learning algorithms to enhance their effectiveness. Additionally, we implemented several feature engineering techniques to enhance the performance of the voting and stacking methods. A benchmarking analysis was also undertaken to juxtapose the performance of various types of optimized methods on the Apelin agonists database.

## 2 Material and methods

**FIGURE 1:**
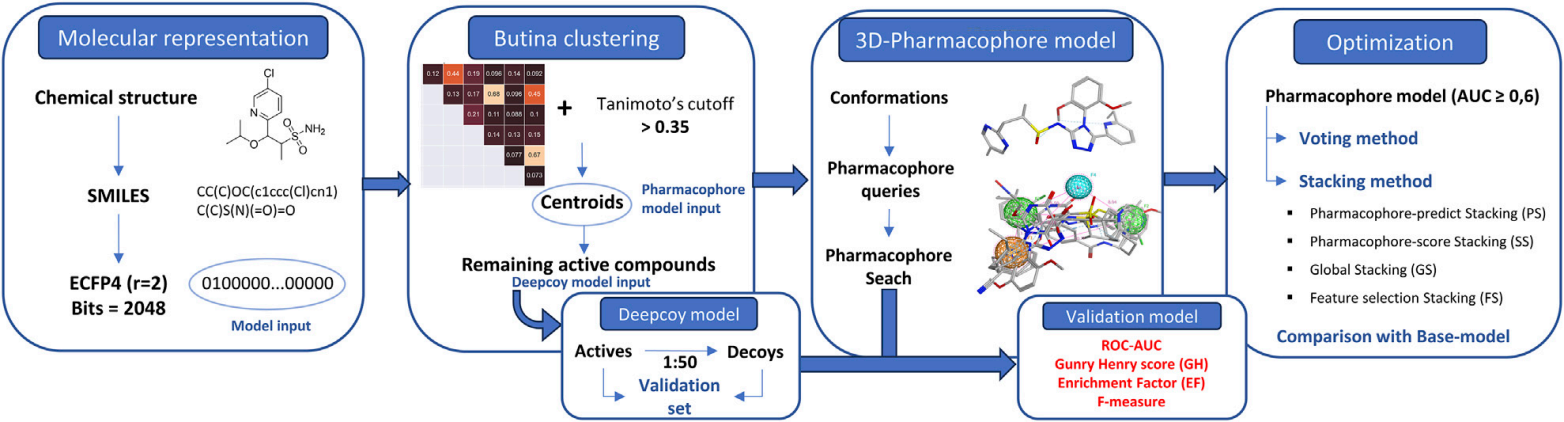
Summary of the entire research process.

The entirety of the research process is encapsulated in Figure 1.

### 2.1 Data preparation

A total of 6,944 compounds procured from papers and patents (Supplementary Table S1–Supplementary Material) underwent a rigorous filtration process based on three specific criteria: the presence of human APJ, agonists, and a biological activity EC50 under 100 nM. This refined data was then standardized, with SMILES (Simplified molecular-input line-entry system) converted to Canonical SMILES, EC50 to pEC50, and any duplicate rows were removed. To ensure the drug-likeness of the data, Lipinski’s Rule of Five (RO5) was implemented (Lipinski et al., 2012). This rule is instrumental in screening oral therapeutic agents and selecting structures that comply with more than three rules (violating only one rule).

### 2.2 Implementing of Butina clustering

Butina clustering was employed to discern smaller, yet homogeneous clusters. This method necessitates that the cluster centroid exhibits a higher degree of similarity (beyond a specified threshold) to every other molecule within the same cluster (Butina, 1999). The proposed workflow is demonstrated as follows:

Molecular fingerprints, specifically extended-connectivity fingerprints (ECFP4) (Rogers and Hahn, 2010) were derived from Canonical SMILESrepresentations using the RDKit package.

The Tanimoto coefficient (Tc) (Chung et al., 2019), given by the formula $Tc=\frac{c}{a+b-c}$ where ‘a’ and ‘b’ represent the number of bits in molecular fingerprints A and B respectively, and ‘c’ is the number of bits shared between A and B, was employed to calculate the pairwise similarity between two fingerprints. This calculation resulted in the formation of an upper triangle similarity matrix, which provides a comprehensive overview of the similarities between different molecular structures. Potential cluster centroids, characterized by having the largest number of neighbors, were identified through a process of sorting the molecules based on their number of neighbors in descending order.

The clustering stage was conducted based on the exclusion spheres method. Here, molecules exhibiting a minimum similarity above a specified cut-off (Tanimoto above 0.35) (Bender et al., 2021) were grouped together into clusters. Each molecule identified as a member of a cluster was flagged and subsequently removed from further comparisons. Molecules that remained unflagged at the end of the clustering process were designated as singletons. These singletons may have neighbors at the given Tanimoto similarity index, but these neighbors would have been excluded by a stronger cluster centroid. Centroids collected from clustering would be used as a training dataset and the remaining actives were used in the decoy generation process.

Table 1 referenced the ButinaClustering algorithm. Where M is a set of M molecules, denoted as $M=\left\{m_{1},\ldots ,m_{M}\right\}$ . Each molecule is associated with a unique fingerprint, collectively represented as the set F . The Tanimoto threshold for similarity between molecules is represented by τ . A triangular matrix T is used to store pairwise Tanimoto coefficients. For each molecule $m_{i}$ , $N_{i}$ represents the number of its neighbors that meet the threshold τ . After the execution of the algorithm, a set of clusters $C=\left\{C_{1},\ldots ,C_{K}\right\}$ is obtained. The centroids of these clusters are represented as $Z=\left\{z_{1},\ldots ,z_{K}\right\}$ . Lastly, a flag set S is used to track which molecules have been assigned to a cluster.

**TABLE 1:** Butina clustering algorithm.

**Algorithm 1** Butina clustering algorithm
1: **Input:** M , F,τ
2: **Output:** C,Z
3: **procedure** BUTINACLUSTERING ( $\left.M,F,\tau \right)$
4: T← TriangularMatrix ( M )
5: **for** i=1 to M **do**
6: **for** j=i+1 to M **do**
7: $T_{ij}\leftarrow T\left(m_{i},m_{j}\right)$
8: **end for**
9: **end for**
10: $N_{i}\leftarrow \sum_{j\neq i}\left[T_{ij}\geq \tau \right]$
11: Sort M by $N_{i}$ in descending order
12: C←∅ , Z←∅,S←∅
13: **for** each $m_{i}\in M\text{ do}$
14: **if** $m_{i}\notin S$ **then**
15: $C\leftarrow \left\{m_{i}\right\}$ , $S\leftarrow S\cup \left\{m_{i}\right\}$
16: **for all** $m_{j}\in M\backslash \left\{m_{i}\right\}$ **do**
17: **if** $T_{ij}\geq \tau$ and $m_{j}\notin S$ **then**
18: $C\leftarrow C\cup \left\{m_{j}\right\}$ , $S\leftarrow S\cup \left\{m_{j}\right\}$
19: **end if**
20: **end for**
21: $C\leftarrow C\cup \left\{C\right\}$ , $Z\leftarrow Z\cup \left\{m_{i}\right\}$
22: **end if**
23: **end for**
24: **return** C,Z
25: **end procedure**

### 2.3 Generating decoys

Decoys were systematically generated using DeepCoy, strategically chosen to mirror the chemical properties of active molecules. Simultaneously, a deliberate mismatch in chemical structure was introduced to mitigate the risk of false negative bias. DeepCoy employed an extensive array of over 25 physicochemical properties, including molecular weight, the number of rotating bonds, the total number of hydrogen donor and acceptor groups, logP, polar surface area, and the sp^3^ fraction of carbon atoms, among others. This comprehensive approach, coupled with a high frequency of repetitions, facilitated the creation of decoys that were challenging to differentiate from active molecules. A critical aspect of the DeepCoy model was its ability to mitigate three prevalent biases in virtual screening datasets: artificial enrichment, analogue bias, and false negative bias. This highlights the significant impact of advanced modeling techniques in enhancing the accuracy and reliability of virtual screening processes. The active molecules were utilized to generate decoys at an imbalanced ratio of 1:50, or 2% (1 active to 50 decoys) (Chaput et al., 2016). This approach was designed to closely emulate the screening process in a real-world setting. The generated decoys were subsequently amalgamated with the active molecules to establish a validation set, which was instrumental in evaluating the performance of the model.

### 2.4 3D-pharmacophore development

The development of a pharmacophore model was undertaken utilizing MOE 2015.10. The initial phase involved the calculation of all potential conformations for each structure, achieved by determining the low-energy conformations of a set of molecules. These conformations were subsequently employed to generate pharmacophore queries that exhibited substantial overlaps in the majority of the active molecules, while maintaining a clear distinction from the inactive ones. The ensuing step involved the execution of a “global search validation”, an automated pipeline designed to validate hundreds of pharmacophore models. This process identified hits that were both active and inactive through the pharmacophore search in MOE. The final results were automatically evaluated using a Python script. This comprehensive and systematic approach underscores the nature of pharmacophore model development and highlights the potential impact of such models on pharmaceutical research.

### 2.5 Optimization

#### 2.5.1 Voting method

The study presents the “global voting” technique, that is the combination of the common hits approach method (Wieder et al., 2017) and ensemble voting method. The “global voting” algorithm consists of three steps. In step 1, top-performing pharmacophore models were selected based on an Area Under the Curve (AUC) threshold. Step 2 involves the construction of the voting model using the inputs of the best pharmacophore models identified in Step 1. Finally, step 3 entailed the validation of the voting model based on the majority of votes (active/inactive).

Table 2 referenced the global voting algorithm. Where P is denoted as a set of all pharmacophore models. The Area Under the Curve (AUC) threshold is represented by θ . A subset, $P_{\text{top}},$ is derived from P , and it includes models with an AUC that is greater than or equal to θ . A voting model, ${\;V}_{\text{model}}$ , is created from these top-performing models in $P_{\text{top}}$ . Lastly, V is a function that creates this voting model from a given set of models.

**TABLE 2:** The global voting algorithm.

**Algorithm 2** global voting algorithm
**Input:** Pharmacophore models set P , AUC threshold θ
**Output:** Voting model $V_{\text{model}}$
$P_{\text{top}}\leftarrow \left\{p\in P\left|\text{AUC}\right.\left(p\right)\geq \theta \right\}$
$V_{\text{model}}\leftarrow V\left(P_{\text{top}}\right)$
**return** $V_{\text{model}}$

#### 2.5.2 Stacking method

The study presents the “global stacking” technique, an innovative adaptation of David’s Stacking algorithm from “stacked generalization” (Wolpert, 1992), tailored specifically to pharmacophore models. Stacking amalgamates predictions from multiple models operating on the same dataset, typically divided into two tiers: level-0 and level-1 models. Level-0 model, or base model, learns directly from the dataset and generates predictions for the level-1 model. The level-1 model, or meta-model, learns from the predictions of the base model.

XGBoost, with its robust capabilities including high efficiency, scalability, intrinsic handling of missing data, regularization features to avert overfitting, customizable modeling options, and inherent cross-validation, is increasingly leveraged in drug discovery, significantly advancing the field by enabling precise predictive modeling and streamlining the identification of novel therapeutic agents. The recent studies by Chen et al. (Chen et al., 2020) and Fang et al. (Fang et al., 2023) also incorporated XGBoost as a training model, showcasing its widespread adoption and effectiveness in the field. For these reasons, we opted for the XGBoost algorithm (Chen and Guestrin, 2016) as the base model. The global stacking technique is a multi-step process that starts with step 1 by selecting top-performing pharmacophore models based on an AUC threshold. In step 2, a new dataset is created using the outputs (predictions/rescores) from these selected models, which then serve as base models for training meta-models. This step utilizes different types of data features for stacking optimization: pharmacophore-predict stacking uses binary “prediction (0/1)" data, pharmacophore-score stacking employs “rescore” data, best global stacking combines both “prediction” and “rescore”, and feature selection stacking uses a feature selection algorithm to optimize “prediction” and “rescore”, step 3 involves training a meta-model using the XGBoost algorithm, where the base model inputs are the pharmacophore models selected in step 1. This meta-model is then re-validated to assess its performance, completing the global stacking process. Table 3 referenced the global stacking algorithm. Where P represents the set of all pharmacophore models and θ is the Area Under the Curve (AUC) threshold. Models from P with AUC greater than or equal to θ form a subset $P_{\text{top}}$ . The outputs of $P_{\text{top}}$ are used to construct a new dataset ${\;D}_{\text{new}}$ . An XGBoost algorithm is applied on $D_{\text{new}\;}$ to train a meta-model $M_{\text{meta}}$ . E is a function that extracts the outputs of the top-performing models into a new dataset, and X is a function that trains the meta-model using the XGBoost algorithm.

**TABLE 3:** The **g**lobal stacking algorithm.

**Algorithm 3** global stacking algorithm
**Input:** Pharmacophore models set P , AUC threshold θ
**Output:** Meta-model $M_{\text{meta}}$
$P_{\text{top}}\leftarrow \left\{p\in P\left|\text{AUC}\right.\left(p\right)\geq \theta \right\}$
$D_{\text{new}}\leftarrow E\left(P_{\text{top}}\right)$
$M_{\text{meta}}\leftarrow X\left(D_{\text{new}}\right)$
**return** $M_{\text{meta}}$

### 2.6 Assessment

Before a pharmacophore model is used in virtual screening, it is crucial to validate it. The model’s quality can be assessed using the Receiver Operating Characteristic (ROC) curve and the AUC. The ROC curve shows the model’s ability to distinguish between active and inactive compounds by plotting the true positive rate against the false positive rate. If the curve is sharp and flat, it means the model ranks active compounds higher than inactive ones. The AUC is a measure of the pharmacophore’s performance and is useful when evaluating multiple models. The AUC can range from 0 to 1, with 1 representing an ideal case where all active compounds are correctly identified first, 0 indicating all inactive compounds are incorrectly classified as active, and 0.5 representing a random state (Fawcett, 2006). Alongside validation metrics, the performance of the model is also evaluated using the statistical parameters of Güner-Henry (GH) (Vyas et al., 2013), enrichment factor (EF) (Sanders et al., 2012), and F-measure (Métivier et al., 2018). The F1 score is the harmonic mean of precision and recall. Recall quantifies the model’s ability to correctly identify all relevant instances (active compounds), aiming to reduce false negatives. On the other hand, precision assesses the accuracy of the model’s positive predictions (active compounds), with an emphasis on reducing false positives. In the context of pharmacophore model assessment, where decoys vastly outnumber active compounds, the F1 score is essential for evaluating performance in imbalanced datasets. This metric spans from 0, denoting poor performance, to 1, indicating perfection. The GH score, which ranges from 0 (representing a null model) to 1 (representing an ideal model), is deemed acceptable if it is higher than 0.7. Conceptually, EF score measures the number of active compounds found in the “early recognition” portion, calculated by determining the ratio of the number of true positive compounds (active compounds) found within a certain percentage of the top-ranked screened compounds to the expected number based on random selection (Truchon and Bayly, 2007).

In an ideal scenario, the model would identify all active compounds without any inactive ones, resulting in a steep ROC curve slope, a high AUC value, a high EF value, and the maximum GH and F1 score values, which are 1. The equations defining GH, F1 score, and EF value are delineated in Eqs 1–3, respectively.

$$\text{GH}=\left(\frac{3}{4}\times \text{Precision}+\frac{1}{4}\times \text{Recall}\right)\times \text{Specificity}$$ (1)

$$F1\text{ score}=\frac{2\times \text{Precision}\times \text{Recall}}{\text{Precision}+\text{Recall}}$$ (2)

$${\text{EF}}_{\chi \%}=\left(\frac{n_{s}}{N_{s}}\right)\times \left(\frac{N}{n}\right)$$ (3)

Where ‘N_s_’ and ‘N’ represent the total number of molecules present in the χ% and in all database, respectively, while ‘n_s_’, and ‘n’ stand for active molecules in χ% and in all database.

## 3 Result

### 3.1 Clustering data using Butina algorithm

**FIGURE 2:**
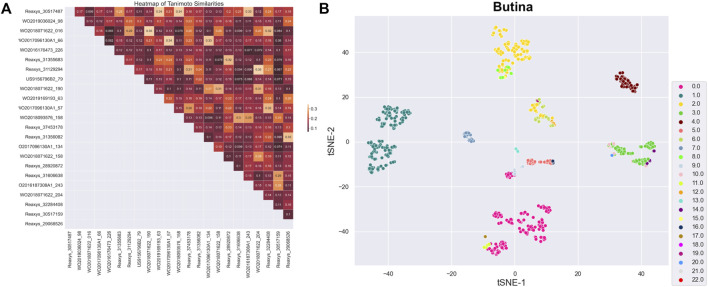
**(A)** Heatmap representing similarity matrix of 23 centroids (the darker the color, the lower the similarity). **(B)** Clustering distribution of 23 centroids.

After preprocessing, 914 active substances were grouped into 23 clusters using the Butina algorithm and a Tanimoto coefficient threshold of 0.35. The Silhouette coefficient, an indicator of clustering performance, was 0.14. The centroids of these clusters were selected for a training set, labeled as set A. To ensure the structural diversity of the selected centroids, a similarity matrix was recalculated for the 23 centroids and visualized as a heatmap chart (Figure 2A). Data dimensionality was reduced to two using principal component analysis (PCA) and t-distributed stochastic neighbor embedding (t-SNE) algorithms, and the clustering distribution was presented using the seaborn library (Figure 2B). While the clustering results were generally satisfactory, with components of each cluster grouped together, there was some overlap between clusters, particularly those with fewer components.

To prevent singletons in the training set, a minimum number of compounds for a viable cluster was specified. The study explored clusters with minimums of 5, 25, 50, and 75 compounds, and the centroids of these clusters were selected for training sets A, B, C, D, and E, respectively (Table 4). Ligand-based pharmacophore models A, B, C, D, and E were built using the five active groups A, B, C, D, and E, respectively. These models were then compared to select the most effective model for virtual screening.

**TABLE 4:** The results of building pharmacophore model.

Results after clustering and building models	Train set				
	Train set A	Train set B	Train set C	Train set D	Train set E
Number of compounds	23	15	8	5	4
Number of conformations	17,789	10,412	4,702	2,387	2047
Number of pharmacophore models	32	166	179	421	370

### 3.2 Validation set

The validation set included 891 active substances remaining after data clustering. Along with 44,313 decoys generated and standardized by the DeepCoy model. The ratio of active to decoy in this set was maintained at 1:50. Finally, decoys generated by DeepCoy achieved a DOE score of 0.045, indicating a close to optimal embedding of actives and decoys in chemical space. Meanwhile, AUC-1NN value of 0.566 demonstrated the greater similarity between the actives and decoys. Besides, the average doppelganger score, a measure of the structural similarity between actives and decoys, was 0.266 for the DeepCoy decoys, while the average maximum doppelganger was 0.469. These results strongly suggested that the decoys generated by DeepCoy should not carry an increased risk of artificial enrichment, analog bias, and false negative bias.

### 3.3 Performance of ligand-based pharmacophore models

This study used five train sets (A, B, C, D, and E) obtained from Butina clustering algorithm by MOE 2015.10 software. Regarding the results illustrated in Table 4, there is an opposite trend when observing the number of substances in the training set and generated pharmacophore models. A possible explanation is that the greater structural diversity in the training set led to the generation of a higher number of conformations. However, the less probability of aligning numerous conformations resulted in the lower models generated.

**FIGURE 3:**
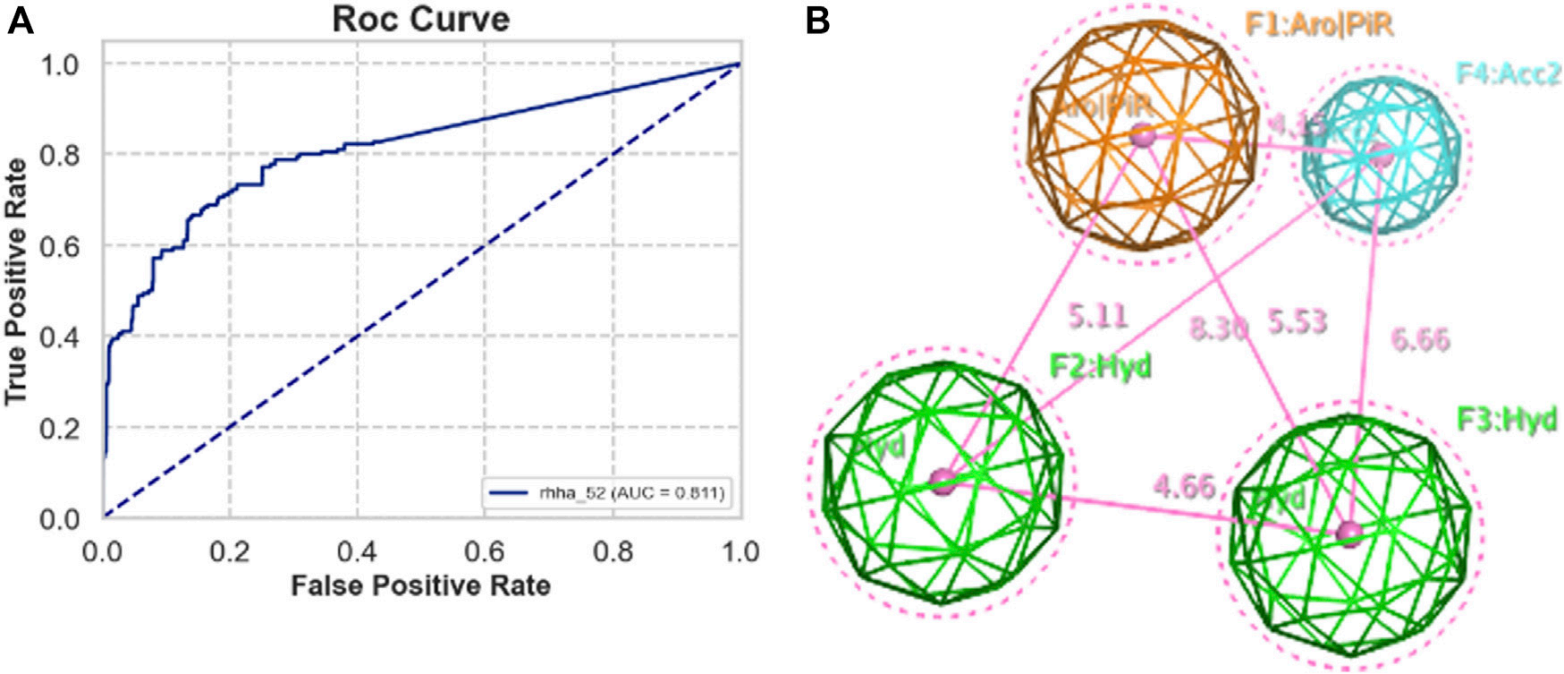
**(A)** ROC curve of RHHa_52 models. **(B)** Best model pharmacophore - RHHa_52 of the train set C. F1 and F2 are hydrophobic groups (**Hyd** with radius of 1.4 Å) and F3 and F4 are hydrogen acceptor groups (**Acc2** with radius of 1.0 Å).

Validation sets, including actives and decoys, were used to assess the performance of pharmacophore models generated by MOE from five train sets A, B, C, D, and E. The standard performance metrics of pharmacophore-based virtual screening included AUC, EF, GH, and F1 score. The priority requirement was as many actives as possible (high TPR) and as few decoys as possible (low FPR), so the area under the ROC curve (AUC) is the priority value. Besides, EF is also a widely used validation metric for assessing the quality of virtual screening protocol to measure how many more actives are found within a defined “early recognition” fraction of the ordered list compared to a random distribution. Based on AUC and EF validation metric, the best pharmacophore model was RHHa_52 from the train set C. This model achieved a high AUC value (0.82) (Figure 3A) and EF1% (19.466), however, GH (0.131) and F1 score (0.071) were very low. The reason was the severe imbalance between the active and the decoy set (ratio 1:50), which led to the extremely low precision of the model, on which the GH and F1 score depended largely. The pharmacophoric feature types of RHHa_52 (Figure 3B) was represented by geometric entities and included two hydrophobic group and two hydrogen bond acceptors. The study set RHHa_52 as the base model to compare the performance of the stacking optimization.

However, the disadvantage of single pharmacophore model is that it uses a single conformation of each substance. In fact, at the binding site, substances could exist in numerous different conformations. Therefore, combining pharmacophore models will be able to solve the problem of diverse conformations in virtual screening.

### 3.4 Optimization of the pharmacophore model

The study conducted ensemble learning method to improve the model’s efficiency by increasing the GH and F1 score. The validation set should be divided according to the stratification principle with a ratio of 80:20 into internal and external validation to ensure generalizability. Three repeats of 10-fold cross-validation were used in internal validation to estimate the model performance.

#### 3.4.1 Voting method

**FIGURE 4:**
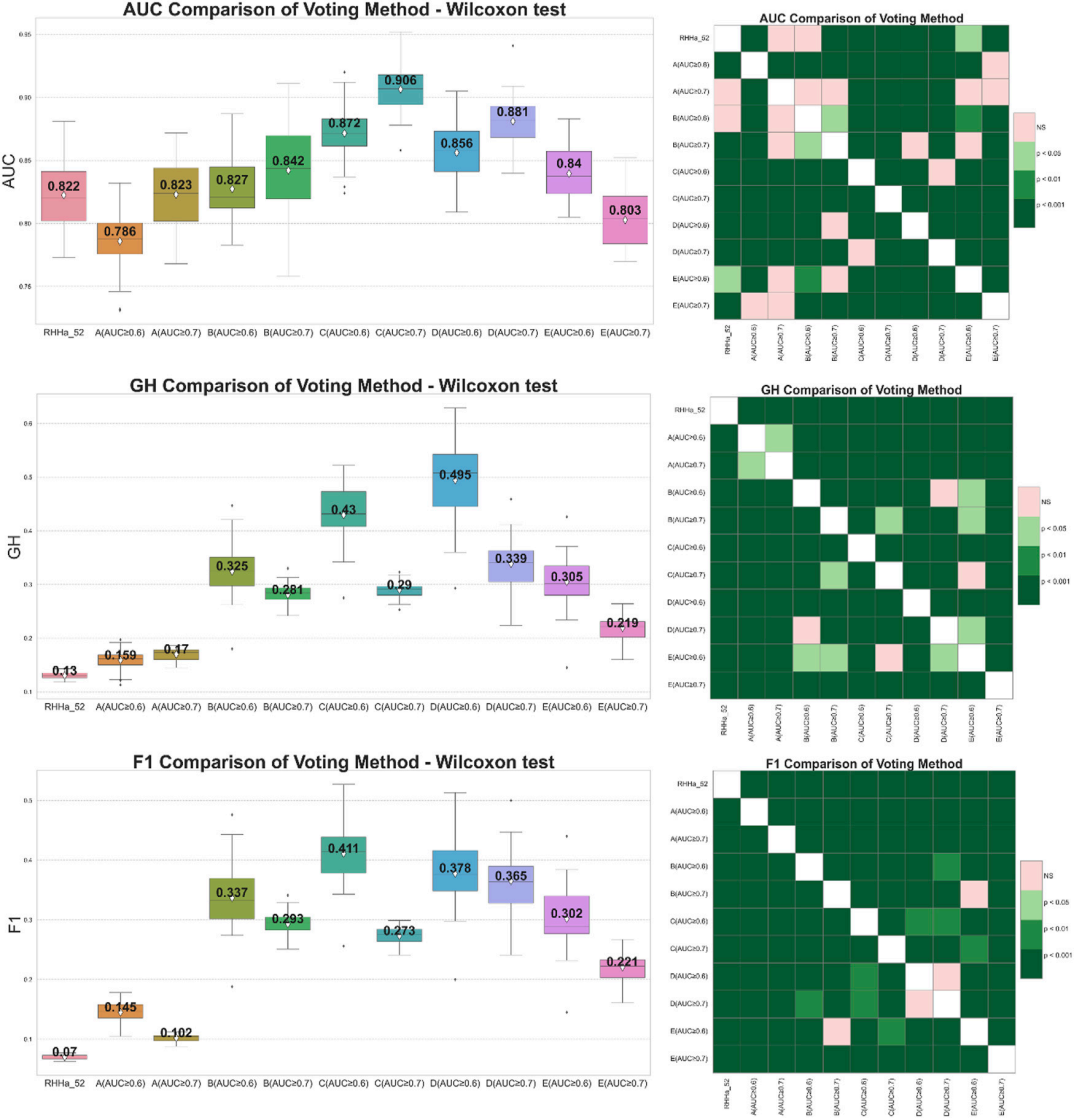
On the left side boxplot describing the results of internal cross-validation and on the right-side Wilcoxon post-hoc test comparing optimized models by global voting algorithm on five training set **(A–E)**. Green representing statistically significant and pink representing no statistically significant difference.

Global voting method was the first filter for this optimization. From the results after global search validation, the study selected two thresholds of AUC, 0.6 and 0.7, in each train set for optimization. The effectiveness of ensemble learning significantly depends on the quality of the individual models it comprises. In ensemble methods like voting or stacking, including poorly performing models can dilute the overall predictive power of the ensemble, leading to suboptimal performance. To mitigate this, we employ an Area Under the Curve of ROC (AUC-ROC) threshold to filter out underperforming models, ensuring that only models with a certain level of predictive ability contribute to the final ensemble decision. AUC thresholds yield varying model counts: ≥0.5 with 124 models, ≥0.6 with 30 models, ≥0.7 with seven models, and ≥0.8 with only two models. An AUC of 0.5 marks the baseline, akin to random guessing. Increasing the threshold filters out weaker models, enhancing the ensemble’s accuracy. However, setting it too high, like at 0.8, might overly constrain the model pool, diminishing diversity and potential effectiveness. Balancing between thresholds of 0.6 and 0.7 could optimize the ensemble’s performance, maintaining a blend of diversity and predictive quality. There were 10 models trained by the global voting method, and the comparison is presented in Figure 4. The results showed that the GH and F1 score of 10 models were better than the base one (RHHa_52). It illustrated a noteworthy contrast in trends: when the number of models increases (AUC ≥0.6), AUC decreased while both F1 score, and GH increased. Specifically, the train set C (AUC ≥0.7) attained the highest AUC index (0.906 ± 0.019), but the GH index (0.29 ± 0.016) and F1 score (0.273 ± 0.015) were relatively low. This phenomenon could be explained by the fact that combining multiple models enhances precision score. By contrast, the recall score witnessed decreases as multiple models must align for an activity prediction. This equilibrium represented a pivotal trade-off essential for model optimization. Besides, the AUC index depended remarkably on sensitivity and specificity compensation, so a decrease in sensitivity led to a decrease in AUC. Conversely, GH and F1 score depended on both accuracy and sensitivity. So that the significant improvement of the accuracy value contributed to the enhancement of these metrics. In general, train set C and train set D with AUC threshold ≥0.6 give better evaluation results than the base model and the remaining models when optimized with the voting algorithm.

#### 3.4.2 Stacking method

**FIGURE 5:**
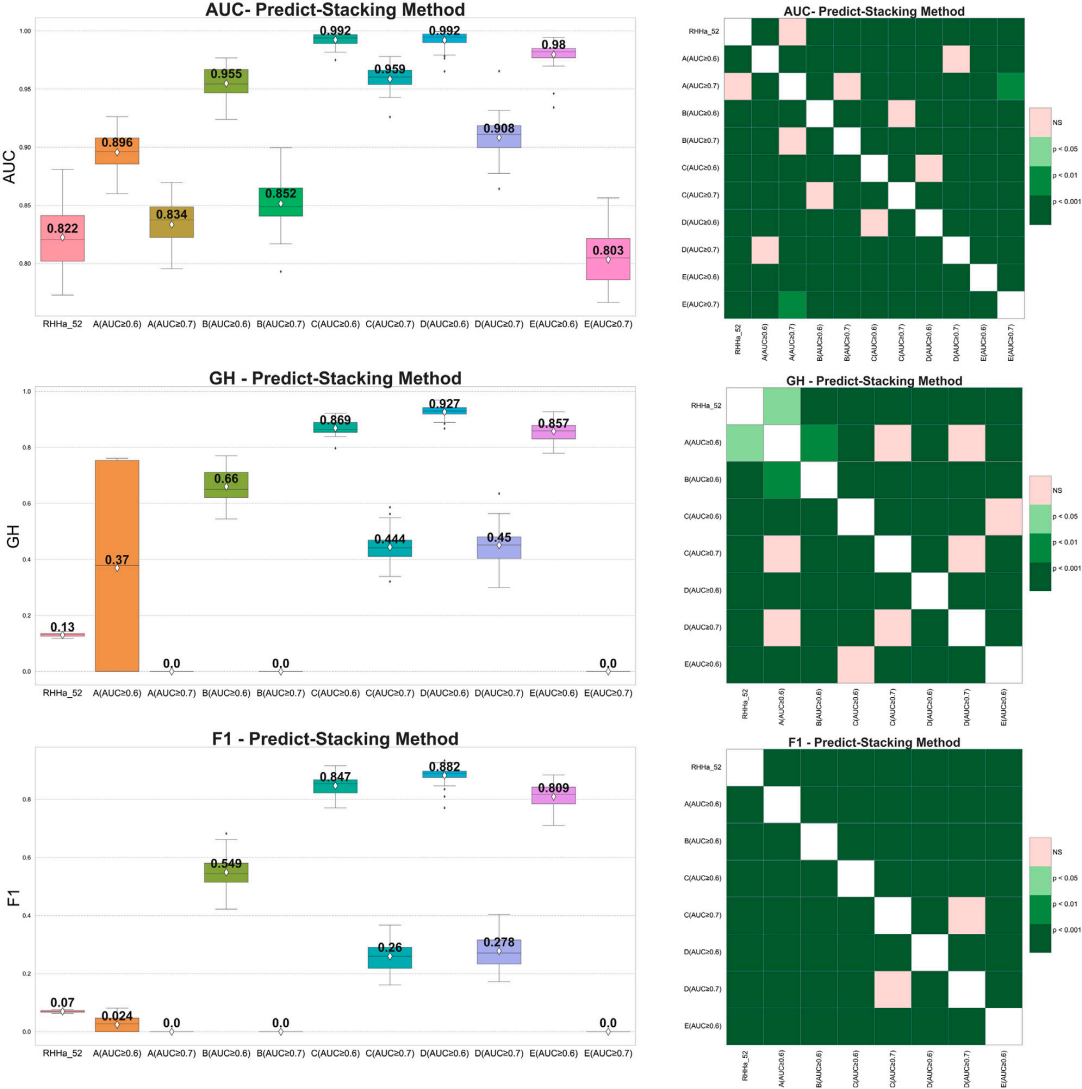
On the left side boxplot describing the results of internal cross-validation and on the right-side Wilcoxon post-hoc test comparing optimized models by pharmacophore-predict stacking algorithm on five training set **(A–E)**. Green representing statistically significant and pink representing no statistically significant difference.

Pharmacophore-predict stacking used the output of the pharmacophore models (prediction 0/1) as the input of the machine learning with the XGBoost algorithm (meta-model). The results showed that the GH and F1 score of 10 models were better than the base one (RHHa_52). Considering Wilcoxon statistics and boxplots, model train set D with threshold AUC ≥0.6 achieved better AUC, GH, and F1 score than the base model and another one. The outcomes of the pharmacophore-predict stacking method are depicted in Figure 5.

Nevertheless, within data sets A, B, and E, where the AUC threshold is set at ≥ 0.7, both F1 score, and GH results yield a value of zero. This can be attributed to the machine learning algorithm’s preference for well-balanced datasets, whereas the dataset for validation was extensively imbalanced (1:50). Additionally, the limited number of input pharmacophore models further compounded the issue. Consequently, the XGBoost algorithm tended to predict all substances as “inactive” to minimize cross-entropy during the training process. As a result, the sensitivity metric reached zero, causing a cascading effect where both F1 score, and GH metrics also registered zero values.

**FIGURE 6:**
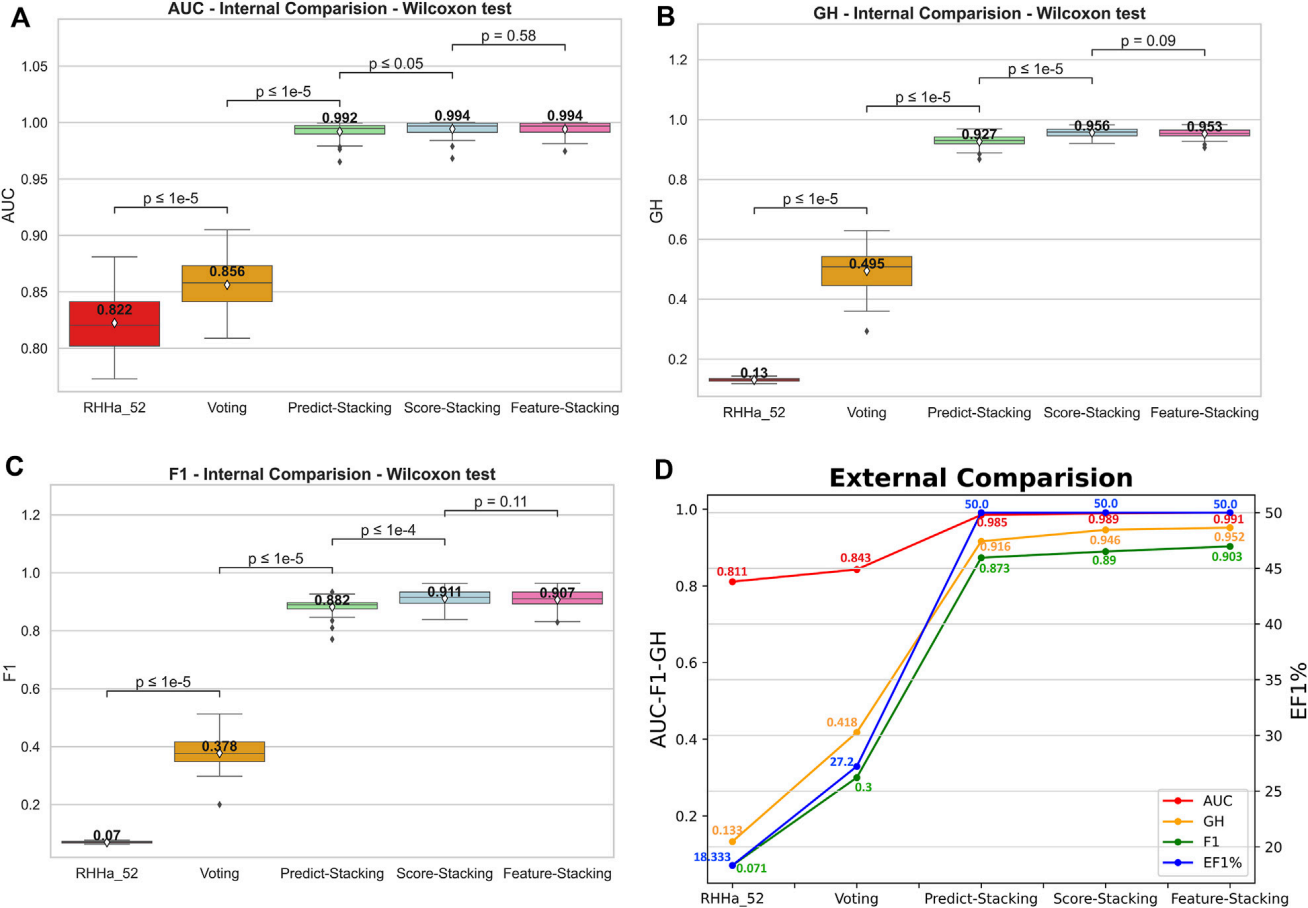
The internal comparison **(A–C)** and the external comparison **(D)** by AUC, GH, and F1 score between base model and 4 Stacking optimal method.

The predictive output of the pharmacophore model is not only a binary variable (0/1) but also a score (rescore). These scores were used to optimize using pharmacophore-score stacking method. When optimizing train set D (threshold AUC ≥0.6), the pharmacophore-score stacking method improved the AUC score by an average of 0.992 ± 0.008 to 0.994 ± 0.007, respectively, increasing from 0.927 ± 0.022 to 0.956 ± 0.015 for GH score and 0.882 ± 0.033 to 0.911 ± 0.031 for F1 score. These results were statistically significant based on the Wilcoxon test (*p* ≤ 0.05). Furthermore, the investigation integrated the prediction (0/1) and score features of the pharmacophore model for optimization through the stacking method, termed global stacking. A drawback of global stacking was the incorporation of numerous features (98 features, including prediction and rescore), potentially leading to the “curse of dimensionality.” To address this issue, the study employed the Random Forest algorithm for feature selection, a process referred to as feature selection stacking. Random Forest selected 44 important elements to optimize. The results showed that the AUC, GH, and F1 score of the feature selection stacking method were not statistically significant by the Wilcoxon test. All the internal-validated comparisons between base model and optimized models were shown in Figures 6A–C.

To illustrate the model’s generalizability, external validation of four optimal methods was depicted in Figure 6D. The outcomes indicated an increase in AUC, GH, and F1 score compared to the base model RHHa_52. Besides, the enrichment factor EF1% also increased from 18.333% (RHHa_52) to 50%. These high values of AUC, EF, GH, and F1 score referred to the good ability of the pharmacophore models to identify active compounds.

To delve deeper into real-world data, we reanalyzed the test set comprising 180 active compounds that were excluded from the training process. The recall or True Positive rate was 83%, signifying that out of 100 pre-identified active compounds, 83 were accurately predicted as active. Additionally, we gathered inactive compound data from US and WO patents, detailed in our repository. Out of 268 compounds, only 31 were incorrectly predicted as active, resulting in a False Positive Rate of 11.57%. These outcomes suggest that our methods are effective with real-world data.

## 4 Discussion

In this investigation, the Butina clustering algorithm facilitated the cultivation of training sets that exhibited a broad spectrum of structural diversity. The ensemble learning methodologies applied, particularly the stacking method, were critically evaluated using an array of performance metrics, namely, AUC, EF1%, GH score, and F1 score. The baseline model, RHHa_52, yielded a moderate AUC of 0.82 and an EF1% of 19.466, as depicted in Figure 3A. However, this model’s utility was compromised by its GH score and F1 score, which were substantially low at 0.131 and 0.071, respectively, signaling an imbalance in the dataset’s active-to-decoy ratio.

A pivotal transformation was observed upon the application of stacking method, which significantly augmented the predictive metrics. A comparative analysis, reinforced by the Wilcoxon test, revealed that stacking method statistically outperformed the base model (*p* ≤ 0.05), culminating in a pronounced increase to an AUC score of 0.994 ± 0.007, a GH score of 0.956 ± 0.015, and an F1 score of 0.911 ± 0.031. These enhancements not only testify to the methodological robustness of stacking method but also its operational efficiency in processing pharmacophore models.

The integration of stacking method within the drug discovery paradigm signifies a methodological advancement that promises to expedite the identification of viable drug candidates through its improved accuracy and efficiency. The statistical validation of this method, as evidenced by the Wilcoxon test, establishes a compelling argument for its preferential use in future pharmacophoric studies.

It is essential to recognize that the current pharmacophore modeling pipeline is not fully automated due to its dependency on the MOE 2015.10 software, which is not compatible with Python scripting. Our strategic objective involves transitioning our pipeline to utilize RDKit for pharmacophore model development, facilitating more seamless integration with our existing framework. Moreover, we aim to broaden our methodology to include structure-based pharmacophore modeling, allowing for the comprehensive extraction of pharmacophore features from proteins. Recognizing the dynamic nature of ligand-protein interactions, our commitment to employing ensemble methods aims to capture a broader spectrum of these interactions, which is expected to significantly enhance the effectiveness of our structure-based pharmacophore models.

## Data Availability

The datasets presented in this study can be found in online repositories. The names of the repository/repositories and accession number(s) can be found in the article/Supplementary Material.
