# Supplementary material for: Integration of the Butina algorithm and ensemble learning strategies for the advancement of a pharmacophore ligand-based model: an in silico investigation of apelin agonists
Source: Front Chem. 2024 Apr 16;12:1382319. doi: 10.3389/fchem.2024.1382319 (PMC11058650; doi:10.3389/fchem.2024.1382319)
Supplement: Supplementary file 1 [file DataSheet1.docx]

Supplementary Material

# Supplementary Data

S 1. Table. APJ Agonists Patents

| **Patent** | **Structure** |
| --- | --- |
| WO2016187308A1 | 1,2,4-triazoles |
| WO2018093576A1 | Alkyl-substituted 1,2,4-triazoles |
| WO2019169193A1 | (2,6-Dimethoxyphenyl)-4*H*-1,2,4-triazole-3-carboxamide |
| US9156796B2 | Benzoimidazole-carboxylic acid amide |
| WO2019036024A1 | 2-[1,1’-Biphenyl]-1*H*-benzo[*D*]imidazoles |
| WO2016176473A1 | 2-substituted-5,6- dimethoxybenzothiazoles |
| WO2017096130A1 | 1,2,5-substituted-6-hydroxy-1,4- dihydropyrimidin-4-one |
| WO2018071622A1 | 5-Amino-3-sulfonylpyridine-2,4-diol |

# Supplementary Figures and Tables

## Supplementary Figures


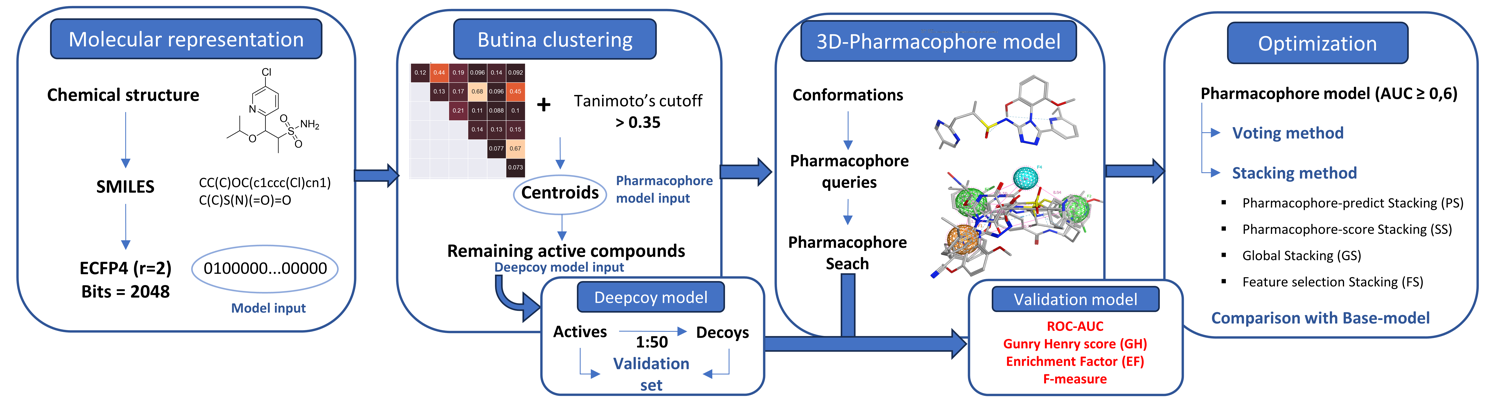


Figure 1. The entire research process.


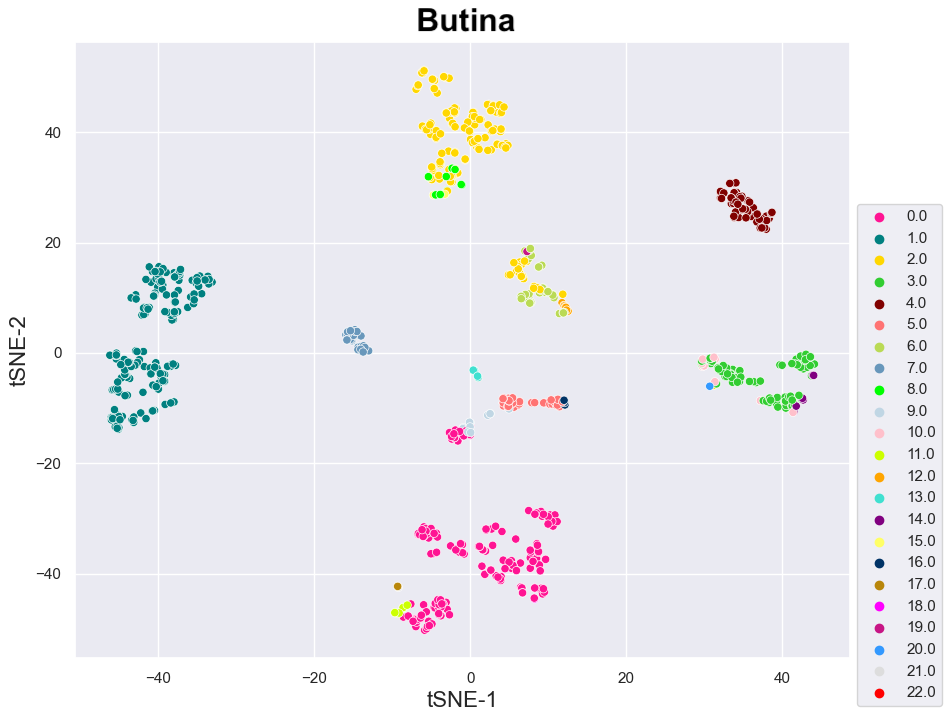


Figure 2(B). Clustering distribution of 23 centroids.


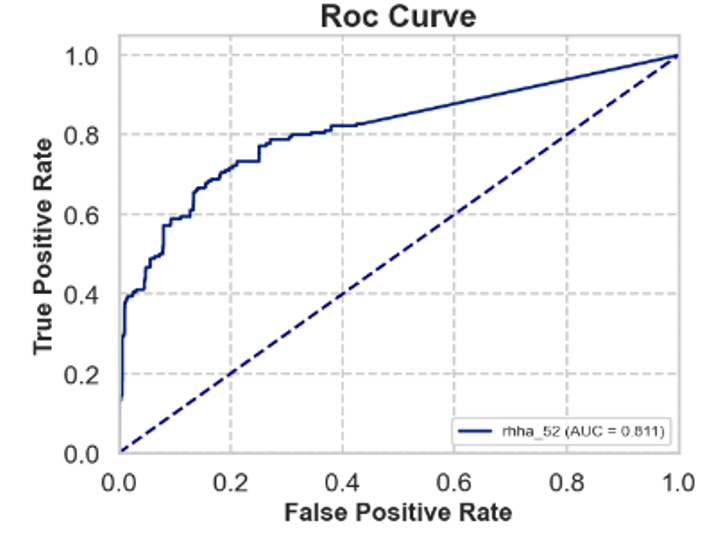


Figure 3(A). ROC curve of RHHa_52 models.


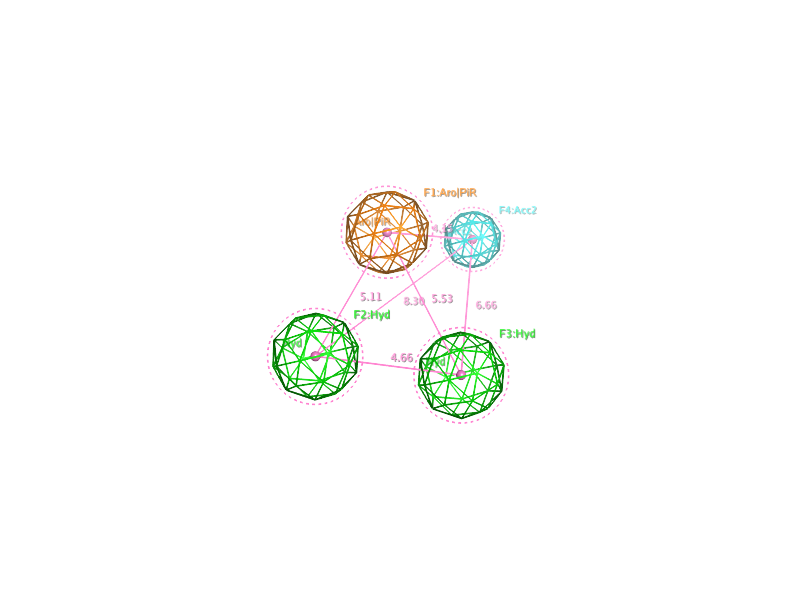


Figure 3(B). Best model Pharmacophore - RHHa_52 of the train set C. F1 and F2 are hydrophobic groups (Hyd with radius of 1.4 Å) and F3 and F4 are hydrogen acceptor groups (Acc2 with radius of 1.0 Å).


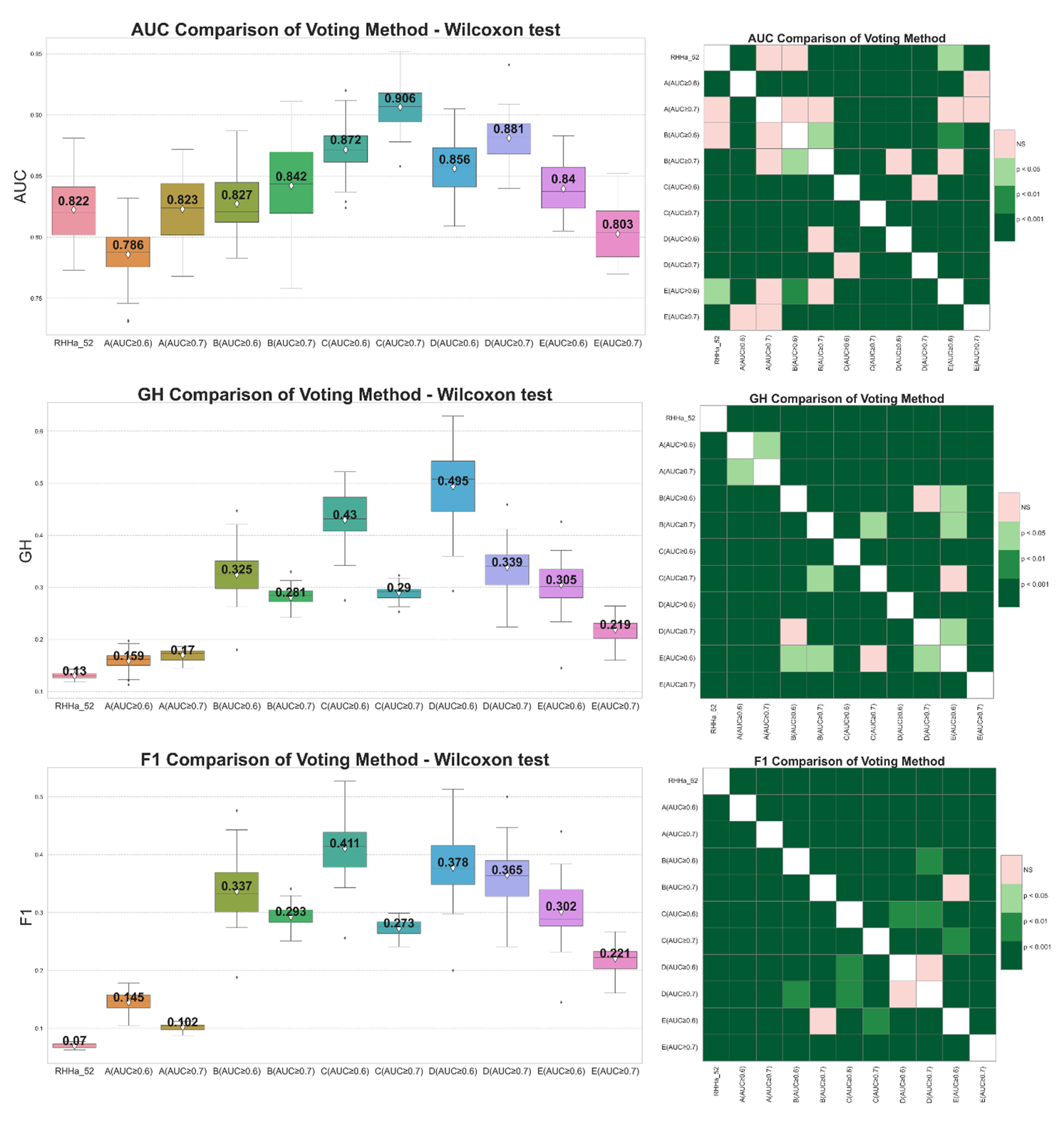


Figure 4. On the left side Boxplot describing the results of internal cross-validation and on the right-side Wilcoxon posthoc test comparing optimized models by Global Voting algorithm on 5 training set (A-E). Green representing statistically significant and pink representing no statistically significant difference.


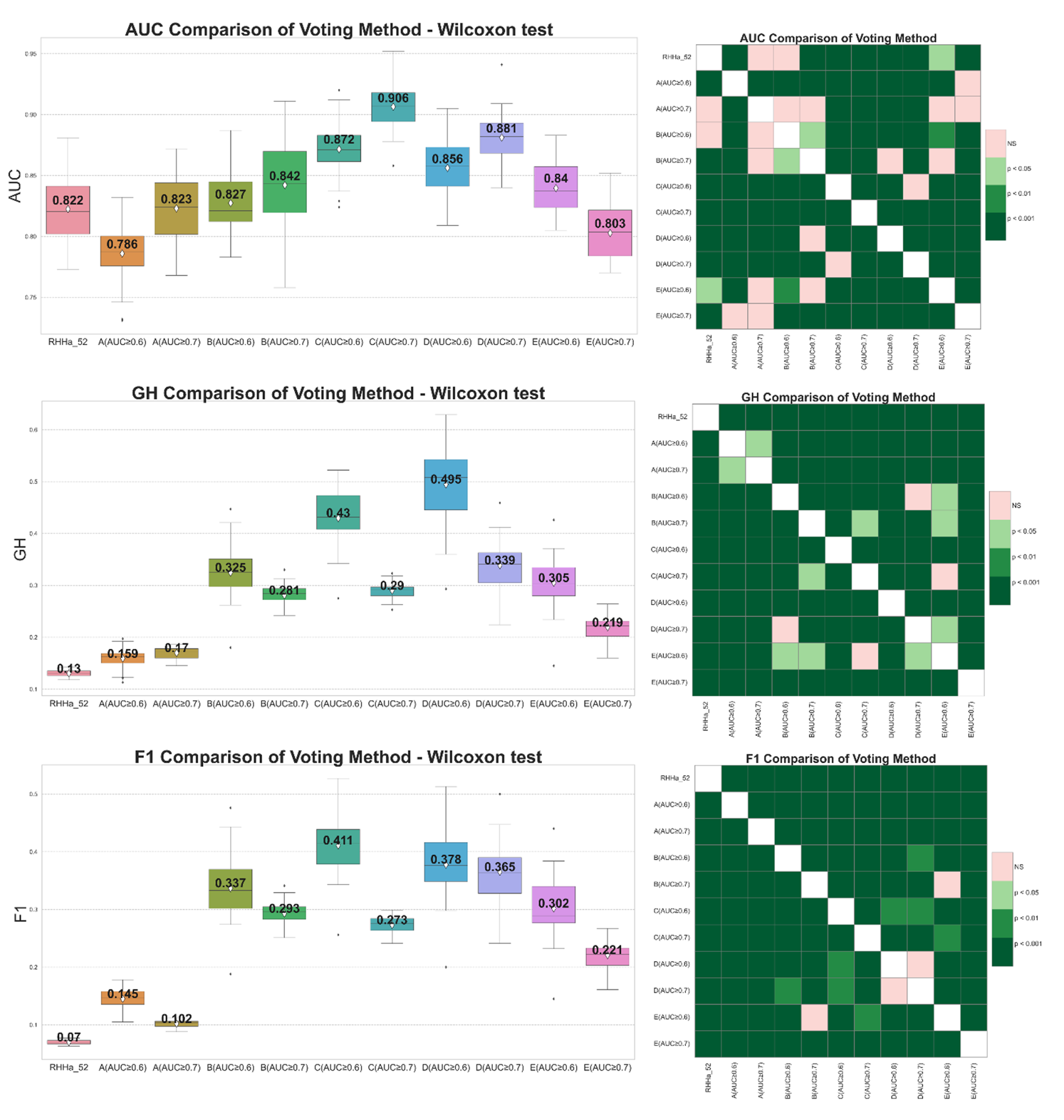


Figure 5. On the left side Boxplot describing the results of internal cross-validation and on the right-side Wilcoxon posthoc test comparing optimized models by pharmacophore-predict Stacking algorithm on 5 training set (A-E). Green representing statistically significant and pink representing no statistically significant difference.


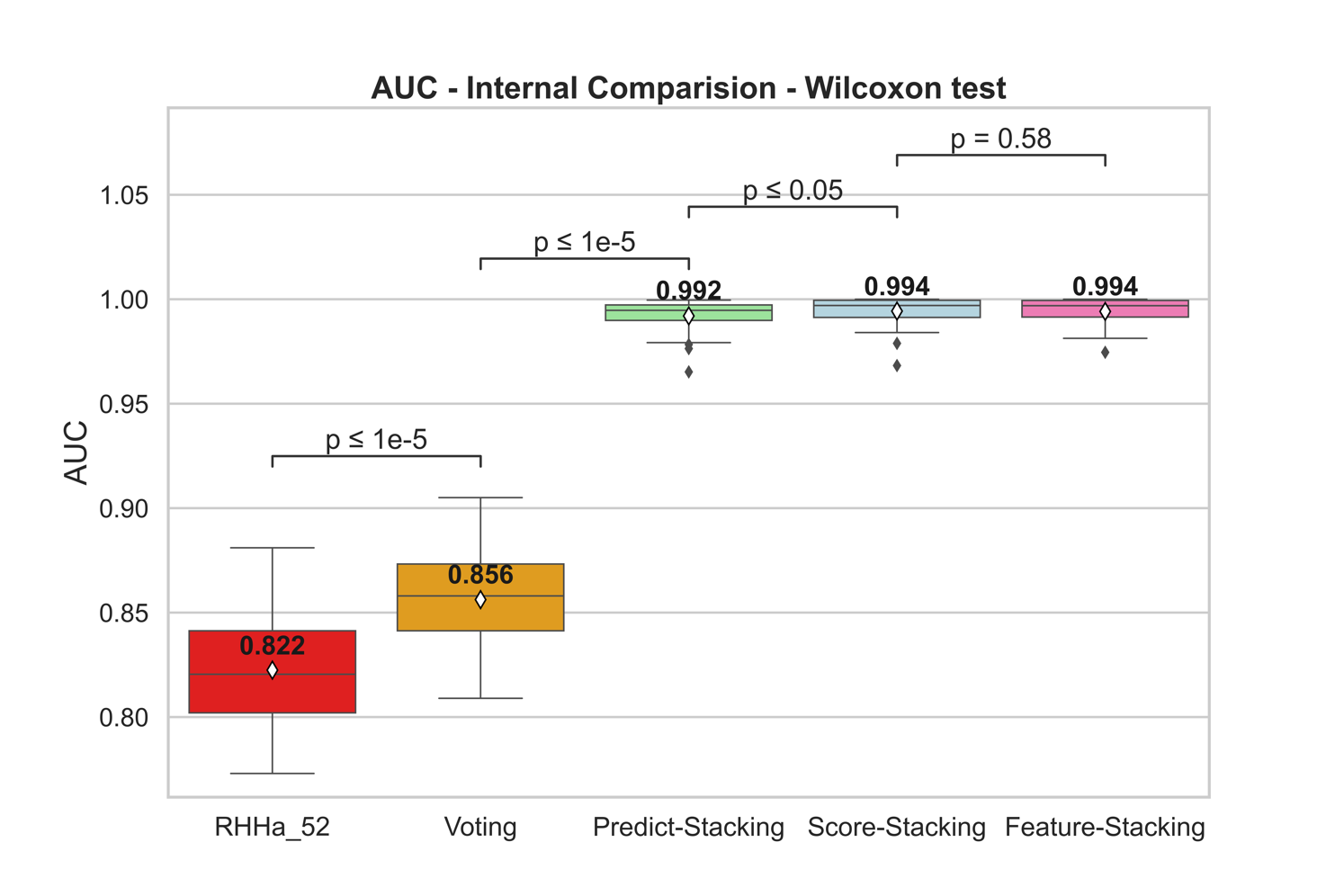


Figure 6(A). The Internal comparison by AUC between base model and 4 Stacking optimal method.


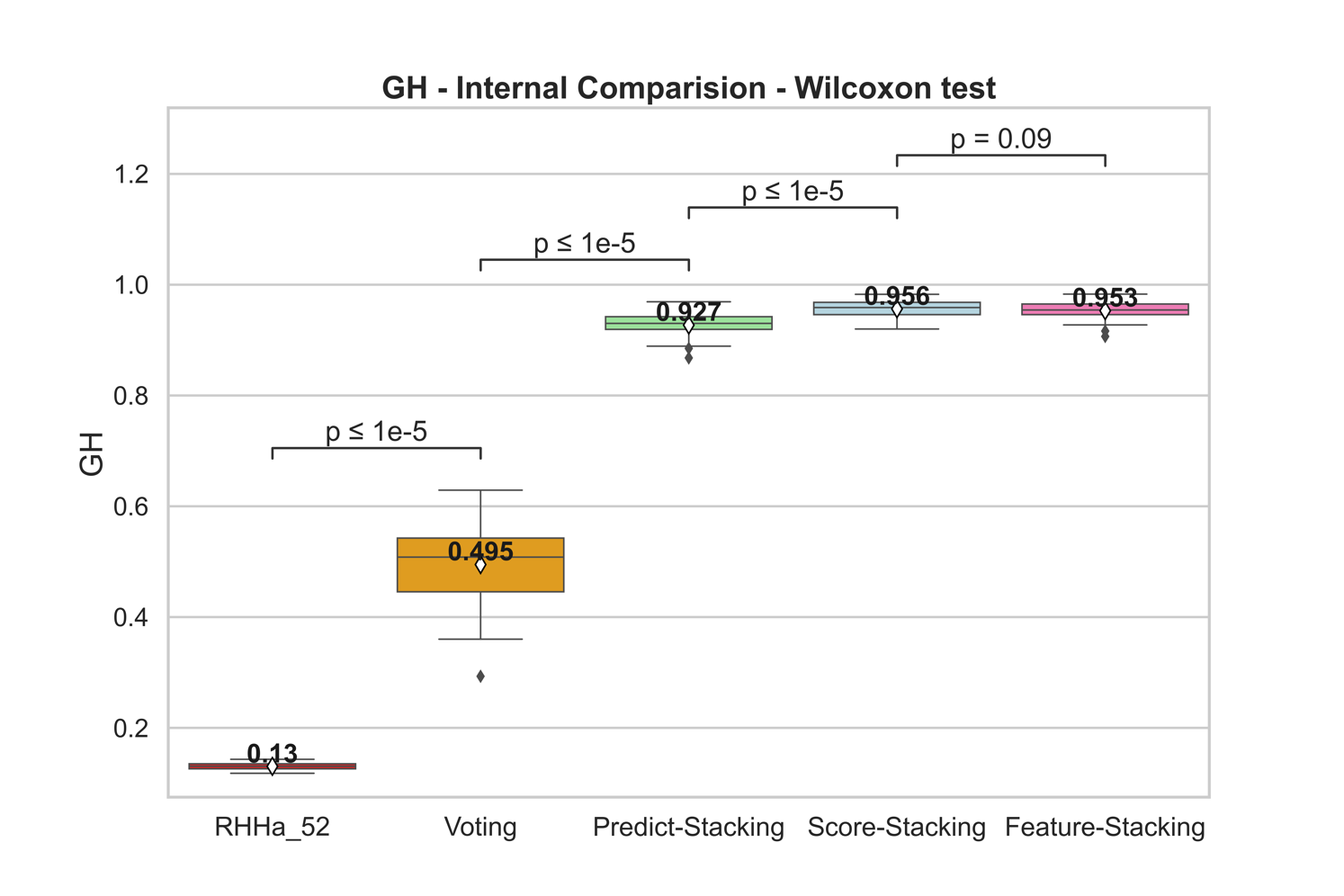


Figure 6(B). The Internal comparison by GH between base model and 4 Stacking optimal method.


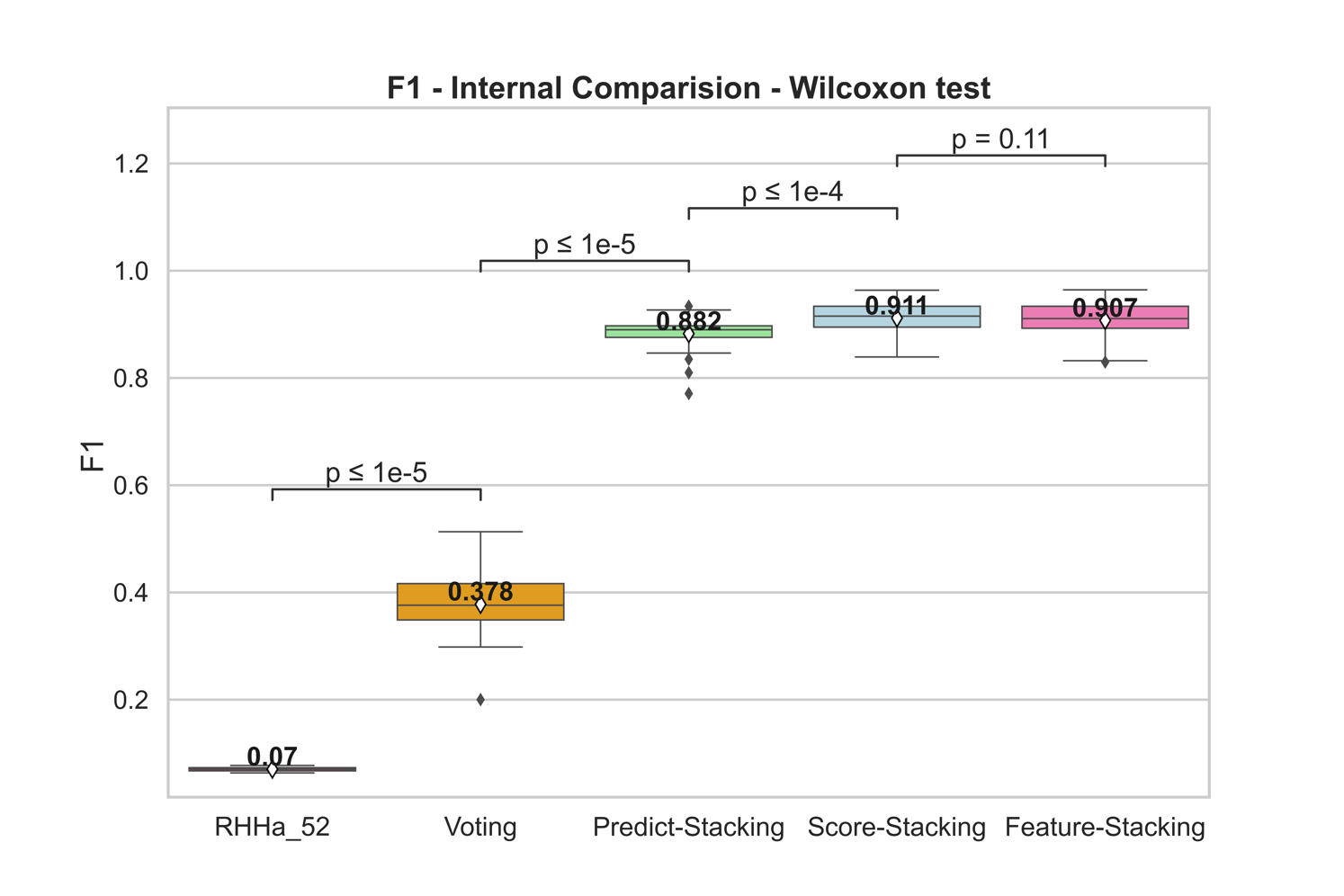


Figure 6(C). The Internal comparison by F1-score between base model and 4 Stacking optimal method.


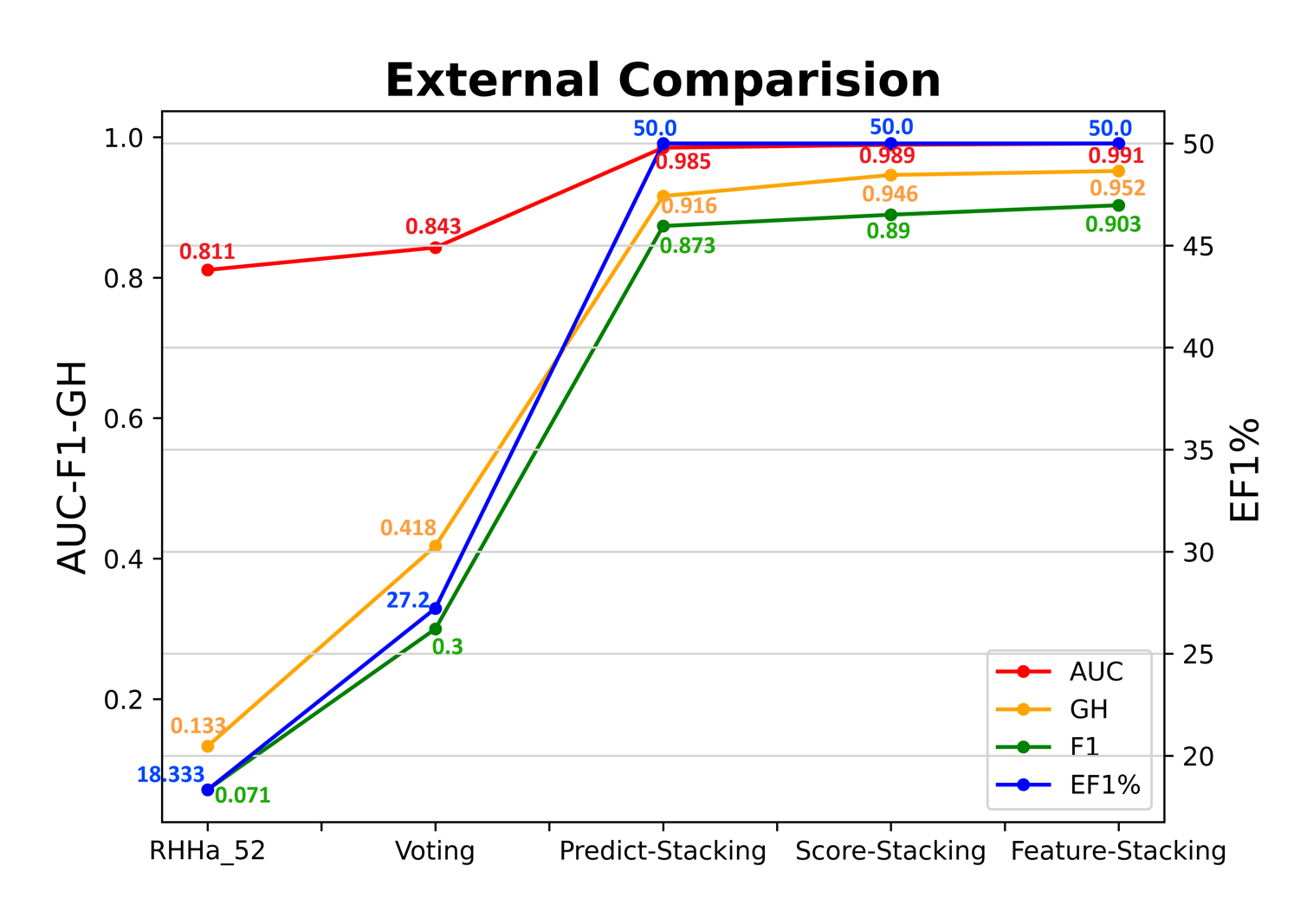


Figure 6(D). The external comparison by AUC, GH, F1-score, EF1% between base model and 4 Stacking optimal method.
